# Pre-intensive care unit use of selective serotonin reuptake inhibitors and mortality in critically ill adults with mental disorders: analysis from the MIMIC-IV database

**DOI:** 10.1038/s41398-023-02487-2

**Published:** 2023-06-06

**Authors:** Wan-Jie Gu, Lu-Ming Zhang, Chun-Mei Wang, Feng-Zhi Zhao, Hai-Yan Yin, Jun Lyu

**Affiliations:** 1 Department of Intensive Care Unit, The First Affiliated Hospital of Jinan University, Guangzhou, Guangdong China; 2 Department of Clinical Research, The First Affiliated Hospital of Jinan University, Guangzhou, Guangdong China; 3 Department of Critical Care Medicine, Affiliated Hospital of Weifang Medical University, Weifang, Shandong China

**Keywords:** Bipolar disorder, Depression, Schizophrenia

## Abstract

Selective serotonin reuptake inhibitors (SSRIs) are the most commonly prescribed drugs for mental disorders in critically ill patients. We performed a retrospective cohort study to investigate the association between pre-ICU use of SSRIs and mortality in critically ill adults with mental disorders. We identified critically ill adults with mental disorders based on the Medical Information Mart in Intensive Care-IV database. The exposure was the use of SSRIs during the period after hospital admission and before ICU admission. The outcome was in-hospital mortality. Time-dependent Cox proportional hazards regression models were used to estimate the adjusted hazard ratio (aHR) with 95% confidence interval (CI). To further test the robustness of the results, we performed propensity score matching and marginal structural Cox model estimated by inverse probability of treatment weighting. The original cohort identified 16601 patients. Of those, 2232 (13.4%) received pre-ICU SSRIs, and 14369 (86.6%) did not. Matched cohort obtained 4406 patients, with 2203 patients in each group (SSRIs users vs. non-users). In the original cohort, pre-ICU use of SSRIs was associated with a 24% increase in the hazard for in-hospital mortality (aHR, 1.24; 95% CI, 1.05–1.46; *P* = 0.010). The results were robust in the matched cohort (aHR, 1.26; 95% CI, 1.02–1.57; *P* = 0.032) and the weighted cohort (aHR, 1.43; 95% CI, 1.32–1.54; *P* < 0.001). Pre-ICU use of SSRIs is associated with an increase in the hazard for in-hospital mortality in critically ill adults with mental disorders.

## Introduction

Mental disorders remain among the top ten leading causes of burden worldwide [1]. In critically ill patients, mental disorders are common before intensive care unit (ICU) admission, during ICU stay, and after discharge [2–4]. Both pre-existing and newly developed mental disorders are associated with unfavorable outcomes in critically ill patients [5–7]. Common mental disorders are typically treated with antidepressants and/or antipsychotics. Of these, selective serotonin reuptake inhibitors (SSRIs) are the first-line pharmacological treatments [8], with approximately one in five critically ill patients receiving them before ICU admission [9]. Previous studies suggested that the use of SSRIs was associated with increased mortality in diabetic patients due to ischemic stroke [10] or among older adults with chronic obstructive pulmonary disease [11]. However, data on the association between the use of SSRIs and outcomes in critically ill patients with mental disorders is scarce.

In 2014, a retrospective study on the topic was conducted using the Medical Information Mart in Intensive Care-II (MIMIC-II) database. The study found that the use of SSRIs prior to ICU admission was associated with increased in-hospital mortality in critically ill patients [9]. However, the results need to be interpreted with caution due to not adjusting for potential confounding factors, such as delirium and coma, which are known to increase mortality in critically ill patients [12, 13]. In the present study, we took into account the two factors and performed the mediation analysis to determine whether the association was mediated by delirium or coma. Furthermore, we used the marginal structural Cox model (MSCM) to adjust the daily Sequential Organ Failure Assessment (SOFA) score to explore the association between pre-ICU use of SSRIs and mortality. Moreover, we utilized the most updated version of MIMIC-IV (version 2.0), which offers more detailed medical records than MIMIC-II [14]. Here, we performed a retrospective cohort study based on data from MIMIC-IV version 2.0 to evaluate the association between pre-ICU use of SSRIs and in-hospital mortality in critically ill adults with mental disorders.

## Methods

### Study design

We employed a large-sample retrospective cohort design to determine the association between pre-ICU use of SSRIs and in-hospital mortality in critically ill adults with mental disorders. The study is based on the MIMIC-IV version 2.0 database, in which data were deidentified and publicly available. It was approved by the institutional review boards of the Beth Israel Deaconess Medical Center and Massachusetts Institute of Technology without a requirement for individual patient informed consent. This study is reported following the Strengthening the Reporting of Observational Studies in Epidemiology statement [15].

### Data source

Data were derived from the latest MIMIC-IV version 2.0 database. MIMIC-IV contains electronic health records from over 50000 patients admitted to the ICUs at the Beth Israel Deaconess Medical Center (Boston, Massachusetts, USA) from 2008 to 2019. Data items included baseline characteristics, illness severity, coexisting conditions, vital signs, laboratory tests, therapeutic interventions, prescribed medications, diagnostic codes, imaging reports, and survival data. The data can be downloaded after completing relevant courses and obtaining a certificate. Data were extracted by structured query language using the patients’ stay_id as the index.

### Patients, exposures, and outcomes

All critically ill adults (age ≥ 18 years) with mental disorders were selected from the MIMIC-IV version 2.0 database. For patients with multiple admissions, only data from the first ICU admission were used. Mental disorders, including depression, anxiety, schizophrenia, bipolar disorders, and other unspecified mental disorders, were identified by the International Classification of Diseases, Ninth Revision (ICD-9) and Tenth Revision (ICD-10) codes with the assistance of a psychiatrist colleague (Supplementary Table 1). The exposure was the use of SSRIs during the period after hospital admission and before ICU admission and extracted from the prescriptions in the MIMIC database. This study included six common SSRIs prescriptions: citalopram, escitalopram, fluoxetine, sertraline, paroxetine, and fluvoxamine. The outcome was in-hospital mortality. Follow-up began with hospital admission and ended at either in-hospital death or hospital discharge. All variables included in this study were missing <2% (Supplementary Fig. 1). Given the low level of missingness, patients with incomplete or missing data were excluded.

### Covariates

We collected patients’ records after ICU admission, including age, sex, weight, admission type, first care unit, Charlson comorbidity index, Simplified Acute Physiology Score (SAPS) II, daily SOFA score, delirium during ICU stay (assessed by Confusion Assessment Method for the ICU) [16], coma during ICU stay (assessed by Richmond Agitation Sedation Scale –4 or −5) [17], therapy at baseline (antibiotic, vasopressors, continuous renal replacement therapy, and invasive mechanical ventilation), coexisting conditions (depression, cerebrovascular disease, dementia, hypertension, myocardial infarct, congestive heart failure, chronic pulmonary disease, diabetes mellitus, renal disease, liver disease, cancer, and sepsis), and medications (other antidepressants, antipsychotics, and Z drugs; Supplementary Table 2). We extracted data on the first vital signs (heart rate, mean arterial pressure, respiratory rate, body temperature, and SpO2) and first laboratory tests (white blood cell, hemoglobin, platelet, potassium, sodium, chloride, bicarbonate, glucose, creatinine, and urea nitrogen).

### Statistical analysis

Continuous variables were expressed as median (interquartile range [IQR]) and were compared by the Mann-Whitney U test. Categorical variables were expressed as numbers (percentages) and were compared by the Chi-square test or Fisher’s exact test. Time-dependent Cox proportional hazards regression models were used to estimate the adjusted hazard ratio (aHR) with 95% confidence interval (CI) for the association between pre-ICU use of SSRIs and mortality. The Schoenfeld residual test was used to check the proportional hazard assumption, and variables with a *P*-value > 0.05 met the assumption. When it is not met, time-varying effects occur. The coxph() function has a tt argument to specify the specific time transformation. In this study, the tt function was applied to the time-varying variables and defined as “tt = function(x, t,…) x * log(t + 20)”, where x is a time-varying variable and t is the analysis time [18]. Based on clinical expertise and previous literature in combination with a statistical perspective, in the multivariate time-dependent Cox proportional hazards regression models, we included the following variables: age, sex, admission type, first care unit, Charlson comorbidity index, depression, sepsis, delirium, coma, SAPS II, SOFA, antibiotic, vasopressors, invasive mechanical ventilation, other antidepressants, antipsychotics, and Z drugs [12, 19–22]. The variance inflation factors (VIFs) were used to examine the presence of multicollinearity among variables.

We performed propensity score matching (PSM) to balance the confounding factors before treatment between the groups. PSM was constructed with a matched cohort of patients with SSRIs and those without SSRIs with similar propensity score values. Specifically, we used a multiple logistic regression model to estimate patients’ propensity scores while applying one-to-one nearest neighbor matching with a caliper width of 0.02 without replacement [23].

In the MSCM, potential variables included age, sex, admission type, first care unit, Charlson comorbidity index, depression, sepsis, delirium, coma, SAPS II, antibiotic, vasopressors, invasive mechanical ventilation, other antidepressants, antipsychotics, and Z drugs. Besides, the daily SOFA score was included in the MSCM as a time-varying confounding factor. Inverse probability of treatment weighting (IPTW) was used to estimate the parameters of MSCM [24, 25]. IPTW applied the inverse of the propensity score as a weight to construct two pseudo-populations using the original population, also known as the weighted cohort [23]. XGBoost (Extreme Gradient Boosting) is an efficient gradient boosting decision tree algorithm that can be used to estimate propensity scores, achieved with the R package “twang” [26]. To reflect the degree of influence of different covariates on the groups or the degree of imbalance between the groups, we applied the XGBoost model to rank the 45 covariates included in this study. The main parameters of the XGBoost model can be found in Supplementary Table 3. The validity of PSM and IPTW was assessed by calculating the standardized mean differences (SMDs), with SMD < 0.1 considered to establish a balanced distribution of confounding factors between the groups [27]. Additionally, we utilized the absolute standardized differences and the distribution balance for the variables before and after weighting to provide the diagnostics for PSM and IPTW [28, 29].

### Subgroup analyses

We performed subgroup analyses to investigate the association between pre-ICU use of SSRIs and mortality in different subgroups. The original population was classified into subgroups based on the following characteristics: age (<65 and ≥65 years), sex (male and female), first care unit (medical/surgical ICU and other ICUs), and population (depression and other mental disorders).

### Further analyses

In the original cohort, we performed several further analyses. First, the mediation analysis to determine whether delirium or coma was the mediator of the association between pre-ICU use of SSRIs and mortality [30]. Second, the class effect analysis (i.e., single drug of SSRIs). Third, the analysis to compare the effect of continuing or stopping SSRIs during ICU stay on mortality.

All statistical analyses were performed using R version 4.1.0 (R Foundation). A two-sided *P*-value < 0.05 was considered to indicate statistical significance.

## Results

### Patient selection

**Fig. 1:**
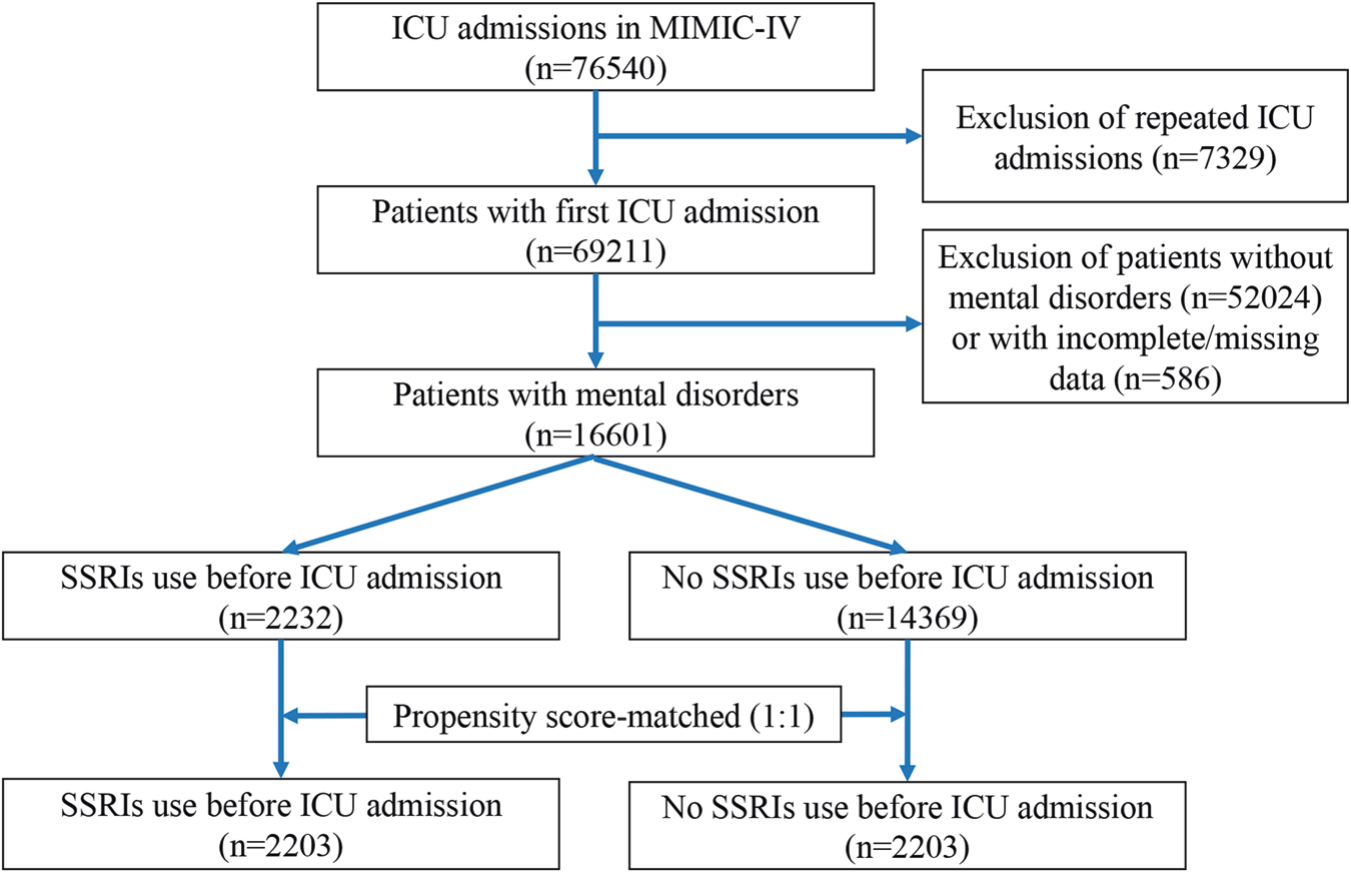
Flow diagram of patient selection. ICU intensive care unit, SSRIs selective serotonin reuptake inhibitors.

Figure 1 shows the process of patient selection. We identified 16601 patients in the original cohort. Of those, 2232 (13.4%) received pre-ICU SSRIs, and 14369 (86.6%) did not. In the matched cohort, we obtained 4406 patients, with 2203 patients in each group. In the weighted cohort, we also obtained two hypothetical populations with a ratio close to 1:1 (Supplementary Table 4).

### Baseline characteristics

Table 1 displays the detailed baseline characteristics. In the original cohort, compared with patients in the non-users group, those in the SSRIs users group were older (median [IQR], 65.0 [55.0, 75.0] years vs. 62.0 [51.0, 73.0] years; *P* < 0.001), had higher Charlson comorbidity index (median [IQR], 6.0 [4.0, 8.0] vs. 5.0 [3.0, 7.0]; *P* < 0.001), were more likely to be receiving antibiotic (1405/2232 [62.9%] vs. 8273/14369 [57.6%]; *P* < 0.001) and invasive mechanical ventilation (587/2232 [26.3%] vs. 3368/14369 [23.4%]; *P* = 0.003), and had higher prevalence of coexisting conditions, including hypertension, myocardial infarct, congestive heart failure, chronic pulmonary disease, diabetes mellitus, and renal disease (all *P* < 0.05). In the matched and weighted cohorts, the imbalances in variables before receiving SSRIs were significantly improved between the groups, such as age, sex, and Charlson comorbidity index, with SMDs <0.1 (Supplementary Fig. 2).

**Table 1:** Baseline patient characteristics in the original and matched cohorts.

Variables	Original cohort			Matched cohort		
	Patients, No. (%)		*P*-value	Patients, No. (%)		*P*-value
	SSRIs users (*n* = 2232)	Non-users (*n* = 14369)		SSRIs users (*n* = 2203)	Non-users (*n* = 2203)	
Age, median (IQR), year	65.0 (55.0, 75.0)	62.0 (51.0, 73.0)	<0.001	65.0 (55.0, 75.0)	66.0 (56.0, 76.0)	0.429
**Sex**						
Male	972 (43.5)	6687 (46.5)	0.009	964 (43.8)	959 (43.5)	0.903
Female	1260 (56.5)	7682 (53.5)		1239 (56.2)	1244 (56.5)	
Weight, kg	79.6 (66.7, 95.6)	78.0 (65.2, 94.1)	0.001	79.6 (66.7, 95.6)	80.1 (67.2, 96.8)	0.273
Charlson comorbidity index, median (IQR)	6.0 (4.0, 8.0)	5.0 (3.0, 7.0)	<0.001	6.0 (4.0, 8.0)	6.0 (4.0, 8.0)	0.595
SAPS II, median (IQR)	33.0 (26.0, 41.0)	31.0 (23.0, 40.0)	<0.001	33.0 (26.0, 41.0)	33.0 (25.0, 42.0)	0.621
SOFA, median (IQR)	2.0 (0.0, 3.0)	1.0 (0.0, 3.0)	<0.001	2.0 (0.0, 3.0)	1.0 (0.0, 3.0)	<.001
**Admission type**						
Emergency	1048 (47.0)	8522 (59.3)	<0.001	1040 (47.2)	993 (45.1)	0.364
Urgent	418 (18.7)	2337 (16.3)		410 (18.6)	428 (19.4)	
Other^a^	766 (34.3)	3510 (24.4)		753 (34.2)	782 (35.5)	
**First care unit**						
Medical/surgical ICU	1360 (60.9)	10580 (73.6)	<0.001	1341 (60.9)	1466 (66.5)	<0.001
Other^b^	872 (39.1)	3789 (26.4)		862 (39.1)	737 (33.5)	
Delirium during ICU stay	529 (23.7)	4157 (28.9)	<0.001	519 (23.6)	643 (29.2)	<0.001
Coma during ICU stay	614 (27.5)	3374 (23.5)	<0.001	603 (27.4)	579 (26.3)	0.434
**Therapy at baseline**						
Antibiotic	1405 (62.9)	8273 (57.6)	<0.001	1382 (62.7)	1319 (59.9)	0.055
Vasopressors	335 (15.0)	2501 (17.4)	0.006	327 (14.8)	415 (18.8)	<0.001
CRRT	19 (0.9)	154 (1.1)	0.400	19 (0.9)	22 (1.0)	0.754
Invasive mechanical ventilation	587 (26.3)	3368 (23.4)	0.003	580 (26.3)	506 (23.0)	0.011
**Coexisting conditions**						
Depression	1766 (79.1)	8769 (61.0)	<0.001	1740 (79.0)	1726 (78.3)	0.633
Cerebrovascular disease	291 (13.0)	1859 (12.9)	0.923	289 (13.1)	316 (14.3)	0.255
Dementia	78 (3.5)	689 (4.8)	0.008	78 (3.5)	67 (3.0)	0.398
Hypertension	949 (42.5)	5711 (39.7)	0.014	941 (42.7)	952 (43.2)	0.761
Myocardial infarct	383 (17.2)	2164 (15.1)	0.011	380 (17.2)	384 (17.4)	0.905
Congestive heart failure	767 (34.4)	3844 (26.8)	<0.001	756 (34.3)	780 (35.4)	0.467
Chronic pulmonary disease	831 (37.2)	4585 (31.9)	<.001	817 (37.1)	853 (38.7)	0.277
Diabetes mellitus	714 (32.0)	4278 (29.8)	0.036	705 (32.0)	739 (33.5)	0.290
Renal disease	529 (23.7)	2836 (19.7)	<0.001	522 (23.7)	538 (24.4)	0.597
Liver disease	299 (13.4)	2072 (14.4)	0.210	294 (13.3)	285 (12.9)	0.721
Cancer	264 (11.8)	1627 (11.3)	0.507	260 (11.8)	275 (12.5)	0.518
Sepsis	922 (41.3)	6703 (46.6)	<0.001	905 (41.1)	1028 (46.7)	<0.001
**Vital signs**, median (IQR)						
Heart rate (beats/min)	83.0 (74.0, 98.0)	88.0 (76.0, 103.0)	<0.001	83.0 (74.0, 98.0)	87.0 (75.0, 101.0)	<0.001
Mean arterial pressure (mm Hg)	81.0 (71.0, 93.0)	84.0 (73.0, 97.0)	<0.001	81.0 (71.0, 93.0)	83.0 (71.0, 95.0)	0.003
Respiratory rate (breaths/min)	18.0 (15.0, 22.0)	18.0 (15.0, 23.0)	<0.001	18.0 (15.0, 22.0)	18.0 (15.0, 23.0)	0.008
Body temperature (°C)	36.7 (36.4, 37.1)	36.8 (36.5, 37.1)	0.001	36.7 (36.4, 37.1)	36.8 (36.5, 37.1)	0.095
SpO2 (%)	98.0 (95.0, 100.0)	98.0 (95.0, 100.0)	0.970	98.0 (95.0, 100.0)	98.0 (95.0, 100.0)	0.297
**Laboratory tests**, median (IQR)						
White blood cell (k/uL)	10.5 (7.5, 14.3)	10.1 (7.1, 14.1)	0.012	10.5 (7.5, 14.3)	10.3 (7.2, 14.4)	0.742
Hemoglobin (g/dL)	10.0 (8.6, 11.5)	10.6 (9.0, 12.2)	<0.001	10.0 (8.6, 11.5)	10.2 (8.9, 11.8)	0.002
Platelet (k/uL)	197.5 (143.0, 266.0)	201.0 (146.0, 265.0)	0.266	197.0 (143.0, 266.0)	198.0 (144.0, 261.0)	0.662
Potassium (mEq/L)	4.1 (3.7, 4.5)	4.1 (3.7, 4.5)	0.015	4.1 (3.7, 4.5)	4.1 (3.7, 4.5)	0.757
Sodium (mEq/L)	138.0 (136.0, 140.0)	139.0 (136.0, 141.0)	<0.001	138.0 (136.0, 140.0)	138.0 (136.0, 141.0)	0.019
Chloride (mEq/L)	103.0 (99.0, 107.0)	104.0 (100.0, 108.0)	0.083	104.0 (99.0, 107.0)	104.0 (99.0, 107.0)	0.948
Bicarbonate (mEq/L)	24.0 (22.0, 27.0)	23.0 (20.0, 26.0)	<0.001	24.0 (22.0, 27.0)	23.0 (21.0, 26.0)	<0.001
Glucose (mg/dL)	122.0 (102.0, 152.0)	122.0 (100.0, 158.0)	0.418	122.0 (102.0, 152.0)	124.0 (102.0, 161.0)	0.028
Creatinine (mg/dL)	0.9 (0.7, 1.4)	0.9 (0.7, 1.4)	0.562	0.9 (0.7, 1.4)	0.9 (0.7, 1.4)	0.153
Urea nitrogen (mg/dL)	18.0 (12.0, 29.0)	18.0 (12.0, 29.0)	0.093	18.0 (12.0, 29.0)	19.0 (12.0, 31.0)	0.021
**Medications**						
Other antidepressants^c^	596 (26.7)	1161 (8.1)	<0.001	572 (26.0)	583 (26.5)	0.732
Antipsychotics	277 (12.4)	524 (3.6)	<0.001	257 (11.7)	221 (10.0)	0.090
Z drugs	85 (3.8)	136 (0.9)	<0.001	74 (3.4)	65 (3.0)	0.490
**Outcomes**						
In-hospital mortality	189 (8.5)	1089 (7.6)	0.155	185 (8.4)	179 (8.1)	0.784
Length of hospital stay (days)	6.1 (4.1, 9.8)	6.1 (3.7, 10.6)	0.319	6.1 (4.1, 9.8)	6.3 (3.9, 10.9)	0.279

*CRRT* continuous renal replacement therapy, *ICU* intensive care unit, *IQR* interquartile range, *SAPS* Simplified Acute Physiology Score, *SOFA* Sequential Organ Failure Assessment, *SSRI* selective serotonin reuptake inhibitor.

^a^Other admission type included elective, observation, and surgical same day admission.

^b^Other first care unit included cardiovascular, trauma, and neurology ICU.

^c^Other antidepressants included monoamine oxidase inhibitors, tricyclics and tetracyclics, selective serotonin reuptake inhibitors, serotonin‐noradrenaline reuptake inhibitors, serotonin partial agonist and reuptake inhibitors, noradrenergic and specific serotoninergic antidepressants, and noradrenaline and dopamine reuptake inhibitors.

### Multivariate analyses

The VIF of each variable was <5, demonstrating the absence of multicollinearity (Supplementary Table 5). Schoenfeld residual plots identify time-varying variables in the original cohort (Supplementary Fig. 3). In the original cohort, multivariable adjustment analysis indicated that pre-ICU use of SSRIs was associated with a 24% increase in the hazard for in-hospital mortality (aHR, 1.24; 95% CI, 1.05–1.46; *P* = 0.010; Table 2).

**Table 2:** Risk of in-hospital mortality associated with pre-ICU use of SSRIs in critically ill adults with mental disorders.

Multivariable analyses	HR	95% CI	*P*-value
Original cohort^a^	1.24	1.05–1.46	0.010
Propensity score-matched cohort^a^	1.26	1.02–1.57	0.032
MSCM in the weighted cohort^b^	1.43	1.32–1.54	<0.001

*CI* confidence interval, *HR* hazard ratio, *MSCM* marginal structural Cox model, *SSRI* selective serotonin reuptake inhibitor.

^a^Adjusted variables included age, sex, admission type, first care unit, Charlson comorbidity index, depression, sepsis, delirium, coma, SAPS II, SOFA, antibiotic, vasopressors, invasive mechanical ventilation, other antidepressants, antipsychotics, and Z drugs.

^b^Adjusted variables included age, sex, admission type, first care unit, Charlson comorbidity index, depression, sepsis, delirium, coma, SAPS II, daily SOFA, antibiotic, vasopressors, invasive mechanical ventilation, other antidepressants, antipsychotics, and Z drugs.

### Propensity score analysis and marginal structural Cox model

**Fig. 2:**
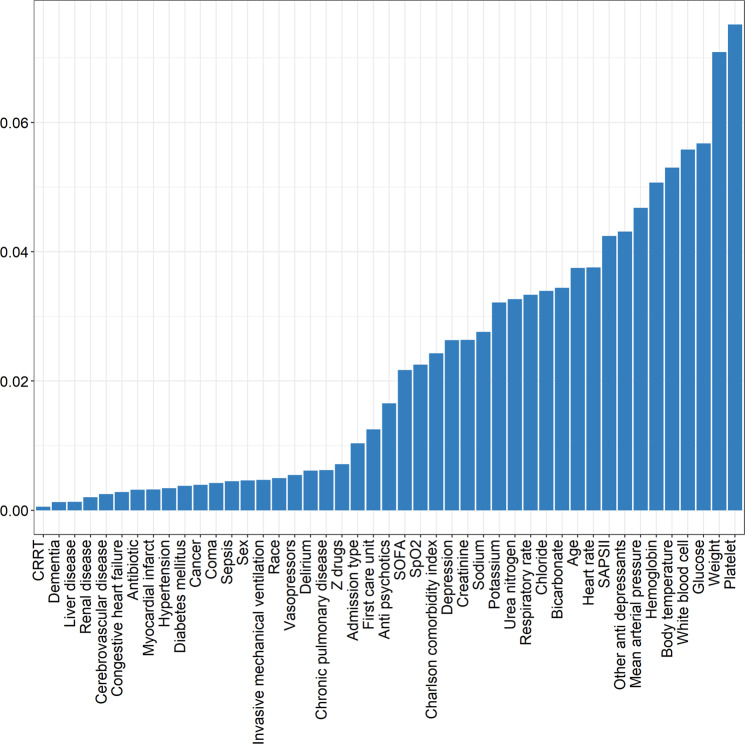
Relative influence factor of covariates. CRRT continuous renal replacement therapy, SAPS Simplified Acute Physiology Score, SOFA Sequential Organ Failure Assessment.

Schoenfeld residual plots identify time-varying variables in the matched cohort (Supplementary Fig. 4). Figure 2 illustrates the extent to which the individual variables in the XGBoost-based IPTW model contribute to the final propensity score. The balance diagnostics for PSM and IPTW were provided in Supplementary Figs. 5–7. In the matched cohort, pre-ICU SSRIs users had a higher risk of in-hospital mortality than non-users (aHR, 1.26; 95% CI, 1.02–1.57; *P* = 0.032; Table 2). In the weighted cohort, MSCM showed that pre-ICU SSRIs use was associated with increased risk of in-hospital mortality (aHR, 1.43; 95% CI, 1.32–1.54; *P* < 0.001; Table 2).

### Subgroup analyses

**Fig. 3:**
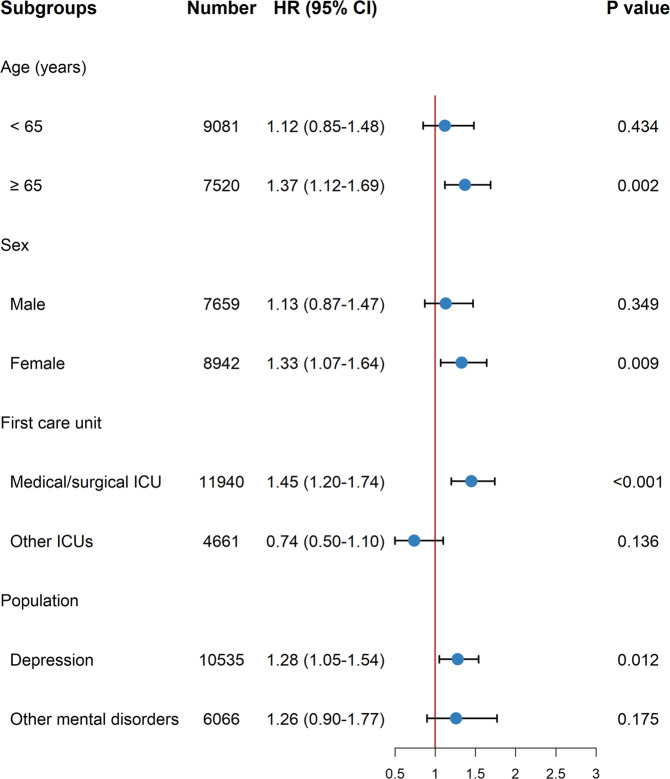
Forest plot for subgroup analyses in the original cohort. CI confidence interval, HR hazard ratio, ICU intensive care unit.

The effect of pre-ICU use of SSRIs on in-hospital mortality varies between different subgroups (Fig. 3). The association was observed only in patients aged ≥65 years (aHR, 1.37; 95% CI, 1.12–1.69; *P* = 0.002), female (aHR, 1.33; 95% CI, 1.07–1.64; *P* = 0.009), admitted into medical/surgical ICU (aHR, 1.45; 95% CI, 1.20–1.74; *P* < 0.001), and with the diagnosis of depression (aHR, 1.28; 95% CI, 1.05–1.54; *P* = 0.012).

### Further analyses

The results of the mediation analysis are shown in Supplementary Table 6. No significant mediation effect was found for either delirium (indirect effect: 0.002, *P* = 0.154) or coma (indirect effect: 0.002, *P* = 0.062). The class effect analysis showed that the increased in-hospital mortality was observed only in fluoxetine (aHR, 1.56; 95% CI, 1.12–2.17; *P* = 0.008; Supplementary Fig. 8). Compared to patients with stopping SSRIs, those with continuing SSRIs had higher in-hospital mortality (aHR, 2.18; 95% CI, 1.59–3.02; *P* < 0.001; Supplementary Table 7).

## Discussion

### Main findings

In this retrospective cohort study, we found that pre-ICU use of SSRIs was associated with an increase in the hazard for in-hospital mortality in critically ill adults with mental disorders. The results were stable in the matched and weighted cohorts.

### Relation with previous evidence

Our study expands on current knowledge regarding the association between the pre-ICU use of SSRIs and mortality in critically ill patients. In a previous retrospective cohort study published in 2014, Ghassemi et al. used data from the MIMIC-II database and found that pre-ICU use of SSRIs was associated with increased in-hospital mortality in critically ill patients (adjusted odds ratio, 1.19; 95% CI, 1.02–1.40) [9]. In line with the previous study, our study found an increased risk of in-hospital mortality associated with pre-ICU use of SSRIs in critically ill patients. However, differences between this study and the previous study should be noted. First, we used the latest MIMIC-IV version 2.0, with more detailed medical records than MIMIC-II. Second, our study is the first to use PSM and IPTW analyses to ensure the robustness of the results. Third, we performed the mediation analysis to determine whether delirium or coma was mediator of the association between pre-ICU use of SSRIs and mortality. Fourth, we performed subgroup analyses and the class effect analysis. Fifth, we further analyzed the effects of continuing versus stopping SSRIs on mortality.

### Possible explanations for findings

Why are critically ill patients with mental disorders receiving pre-ICU use of SSRIs at increased risk of mortality? The underlying mechanisms are far from being fully elucidated and require further exploration. Several potential explanations have been posited. First, SSRIs exert immunosuppressive effects by inhibiting proliferation and enhancing apoptosis of T cells. Gobin et al. observed that SSRIs induce an anti-proliferative effect by reducing T cell proliferation [31]. Besides, SSRIs have been shown to induce T cell apoptosis and decrease activated T cell viability [32]. These immune suppressive changes may increase the susceptibility of critically ill patients to secondary infections and high mortality. In a multicenter study on ICU patients receiving mechanical ventilation, the incidence of multidrug-resistant bacteria and mortality was significantly higher among immunocompromised patients than among non-immunocompromised patients [33]. Another study also found that immunosuppression status was independently associated with mortality in ICU patients with de novo acute hypoxemic respiratory failure treated with noninvasive ventilation [34]. Second, SSRIs may regulate mitochondrial machinery via one important mitochondria-related immune regulator (i.e., mitochondrial DNA) [35]. Circulating mitochondrial DNA has been associated with mortality in critically ill patients [36]. Third, the clinical investigation found that long-term use of SSRIs was associated with increased risks of cerebrovascular disease, cardiovascular disease mortality, and all-cause mortality [37]. A review also summarized the evidence on the potential adverse cardiovascular effects of treatment with SSRIs in patients with geriatric depression [38].

### Implications for clinical practice

Our findings have potential implications for clinical practice, particularly for critically ill patients with pre-existing mental health disorders. A previous systematic review demonstrated that ~19% of adults in ICU suffer from such conditions, making them a significant subgroup [39]. Among the medications prescribed for mental health disorders, SSRIs are commonly used, including for depression, anxiety, and posttraumatic stress disorders [8]. However, there is limited information on the association between the use of SSRIs and outcomes in critically ill patients. A review by Kelly et al. indicated that there might be excess morbidity associated with SSRIs in critically ill patients, but the evidence was primarily derived from low-quality case reports or series [40]. Our study found that pre-ICU use of SSRIs was associated with increased mortality in critically ill adults with mental disorders, highlighting the need to address these gaps in evidence. It is important to note that our findings do not imply that SSRIs should not be used in critically ill patients. Rather, our results may encourage prescribers to consider the potential risk of increased mortality in critically ill patients receiving SSRIs.

Our study provides novel insights into the association between pre-ICU use of SSRIs and mortality. However, we cannot conclude that there is a causal relationship between pre-ICU use of SSRIs and mortality. Even if such a link exists, it is unclear whether any harm is related to the chronic effects of pre-ICU use of SSRIs, SSRIs use during critical illness, or acute SSRIs withdrawal. It is no doubt of interest to critical care physicians that pre-ICU admission use of SSRIs is associated with increased mortality in critical illness; meanwhile, whether critically ill patients should continue or stop using SSRIs during ICU stay is an important question. A retrospective cohort study found that discontinuation of chronic use of SSRIs was associated with a higher risk of antidepressant discontinuation syndrome symptoms in critically ill adults [41]. A recent review summarized the literature on the reinitiation of preadmission neuropsychiatric medications in ICU patients, including SSRIs drugs [42]. La et al. found that early reinitiation of neuropsychiatric medications within 5 days after ICU admission was associated with lighter sedation and less delirium [43]. Conversely, Cucci et al. found no association between early reinitiation within 72 h and delirium after adjustment for baseline differences [44]. Unfortunately, these studies had no data on mortality. Our study showed that continuing SSRIs during ICU stay was associated with increased in-hospital mortality compared to stopping SSRIs, highlighting the importance of this information to help inform clinical decision-making. Overall, evidence on stopping or reinitiating SSRIs and outcomes in critically ill patients is still lacking. More high-quality studies are warranted to help physicians weigh the benefits and harms of SSRIs in critically ill patients.

### Study limitations

Our study has several limitations. First, given that our study is observational, there may be inherent risks, including the possibility of residual and unmeasured confounding that cannot be entirely ruled out. While we attempted to address visible bias by utilizing the PSM and IPTW approach, unmeasured covariates could still be a source of bias. Second, since our retrospective cohort study only established a correlation between exposure and outcomes, further studies using a prospective design are required to establish causality and validate our findings [45]. Third, the use of ICD-9/10 codes to identify mental disorders raises significant reliability concerns, particularly since primary care physicians primarily diagnose mental disorders. As a result, many ICU patients in the MIMIC database may not have received their primary care, resulting in the possibility that the mental disorder codes and medications may not have been documented for these patients in the database. Furthermore, the severity of mental disorders and medication compliance at the time of ICU admission remains unknown. These factors could potentially affect mortality and bias our results. Fourth, delirium/coma may change over time with the severity of the illness. We analyzed the occurrence of delirium/coma during the entire ICU stay rather than the daily status or ICU days with delirium/coma, potentially impacting the results. Fifth, we did not consider pre-ICU mental disorder severity, medication compliance, and the dose/duration of individual SSRIs used in the ICU, and further research is necessary to confirm any association. Lastly, our study only included patients from a single center, which limits its generalizability to other ICUs with varying practices or resources.

## Conclusions

In summary, this retrospective cohort study suggested that pre-ICU use of SSRIs is associated with an increase in the hazard for in-hospital mortality in critically ill adults with mental disorders.

## Supplementary information

Supplemental Material

## Data Availability

The datasets used and/or analyzed during the current study are available from the corresponding author upon reasonable request.

## Code availability

All codes are available from the corresponding author upon reasonable request.
