# Supplementary material for: Pre-intensive care unit use of selective serotonin reuptake inhibitors and mortality in critically ill adults with mental disorders: analysis from the MIMIC-IV database
Source: Transl Psychiatry. 2023 Jun 6;13:187. doi: 10.1038/s41398-023-02487-2 (PMC10241904; doi:10.1038/s41398-023-02487-2)

Supplementary Table 1 ICD codes for mental disorders.

ICD code	ICD version	Long title
**Depression**		
29013	9	Presenile dementia with depressive features
29021	9	Senile dementia with depressive features
29043	9	Vascular dementia, with depressed mood
29620	9	Major depressive affective disorder, single episode, unspecified
29621	9	Major depressive affective disorder, single episode, mild
29622	9	Major depressive affective disorder, single episode, moderate
29623	9	Major depressive affective disorder, single episode, severe, without mention of psychotic behavior
29624	9	Major depressive affective disorder, single episode, severe, specified as with psychotic behavior
29625	9	Major depressive affective disorder, single episode, in partial or unspecified remission
29626	9	Major depressive affective disorder, single episode, in full remission
29630	9	Major depressive affective disorder, recurrent episode, unspecified
29631	9	Major depressive affective disorder, recurrent episode, mild
29632	9	Major depressive affective disorder, recurrent episode, moderate
29633	9	Major depressive affective disorder, recurrent episode, severe, without mention of psychotic behavior
29634	9	Major depressive affective disorder, recurrent episode, severe, specified as with psychotic behavior
29635	9	Major depressive affective disorder, recurrent episode, in partial or unspecified remission
29636	9	Major depressive affective disorder, recurrent episode, in full remission
29650	9	Bipolar I disorder, most recent episode (or current) depressed, unspecified
29651	9	Bipolar I disorder, most recent episode (or current) depressed, mild
29652	9	Bipolar I disorder, most recent episode (or current) depressed, moderate
29653	9	Bipolar I disorder, most recent episode (or current) depressed, severe, without mention of psychotic behavior
29654	9	Bipolar I disorder, most recent episode (or current) depressed, severe, specified as with psychotic behavior
29655	9	Bipolar I disorder, most recent episode (or current) depressed, in partial or unspecified remission
29656	9	Bipolar I disorder, most recent episode (or current) depressed, in full remission
29682	9	Atypical depressive disorder
2980	9	Depressive type psychosis
30112	9	Chronic depressive personality disorder
3090	9	Adjustment disorder with depressed mood
3091	9	Prolonged depressive reaction
30928	9	Adjustment disorder with mixed anxiety and depressed mood
311	9	Depressive disorder, not elsewhere classified
E9287	9	Environmental and accidental causes, mechanism or component of firearm and air gun
F0631	10	Mood disorder due to known physiological condition with depressive features
F0632	10	Mood disorder due to known physiological condition with major depressive-like episode
F251	10	Schizoaffective disorder, depressive type
F313	10	Bipolar disorder, current episode depressed, mild or moderate severity
F3130	10	Bipolar disorder, current episode depressed, mild or moderate severity, unspecified
F3131	10	Bipolar disorder, current episode depressed, mild
F3132	10	Bipolar disorder, current episode depressed, moderate
F314	10	Bipolar disorder, current episode depressed, severe, without psychotic features
F315	10	Bipolar disorder, current episode depressed, severe, with psychotic features
F3175	10	Bipolar disorder, in partial remission, most recent episode depressed
F3176	10	Bipolar disorder, in full remission, most recent episode depressed
F32	10	Major depressive disorder, single episode
F320	10	Major depressive disorder, single episode, mild
F321	10	Major depressive disorder, single episode, moderate
F322	10	Major depressive disorder, single episode, severe without psychotic features
F323	10	Major depressive disorder, single episode, severe with psychotic features
F324	10	Major depressive disorder, single episode, in partial remission
F325	10	Major depressive disorder, single episode, in full remission
F328	10	Other depressive episodes
F3289	10	Other specified depressive episodes
F329	10	Major depressive disorder, single episode, unspecified
F33	10	Major depressive disorder, recurrent
F330	10	Major depressive disorder, recurrent, mild
F331	10	Major depressive disorder, recurrent, moderate
F332	10	Major depressive disorder, recurrent severe without psychotic features
F333	10	Major depressive disorder, recurrent, severe with psychotic symptoms
F334	10	Major depressive disorder, recurrent, in remission
F3340	10	Major depressive disorder, recurrent, in remission, unspecified
F3341	10	Major depressive disorder, recurrent, in partial remission
F3342	10	Major depressive disorder, recurrent, in full remission

F338	10	Other recurrent depressive disorders
F339	10	Major depressive disorder, recurrent, unspecified
F4321	10	Adjustment disorder with depressed mood
F4323	10	Adjustment disorder with mixed anxiety and depressed mood
F530	10	Postpartum depression
**Other mental disorders**		
29384	9	Anxiety disorder in conditions classified elsewhere
29500	9	Simple type schizophrenia, unspecified
29501	9	Simple type schizophrenia, subchronic
29502	9	Simple type schizophrenia, chronic
29503	9	Simple type schizophrenia, subchronic with acute exacerbation
29504	9	Simple type schizophrenia, chronic with acute exacerbation
29505	9	Simple type schizophrenia, in remission
29510	9	Disorganized type schizophrenia, unspecified
29511	9	Disorganized type schizophrenia, subchronic
29512	9	Disorganized type schizophrenia, chronic
29513	9	Disorganized type schizophrenia, subchronic with acute exacerbation
29514	9	Disorganized type schizophrenia, chronic with acute exacerbation
29515	9	Disorganized type schizophrenia, in remission
29520	9	Catatonic type schizophrenia, unspecified
29521	9	Catatonic type schizophrenia, subchronic
29522	9	Catatonic type schizophrenia, chronic
29523	9	Catatonic type schizophrenia, subchronic with acute exacerbation
29524	9	Catatonic type schizophrenia, chronic with acute exacerbation
29525	9	Catatonic type schizophrenia, in remission
29530	9	Paranoid type schizophrenia, unspecified
29531	9	Paranoid type schizophrenia, subchronic
29532	9	Paranoid type schizophrenia, chronic
29533	9	Paranoid type schizophrenia, subchronic with acute exacerbation
29534	9	Paranoid type schizophrenia, chronic with acute exacerbation
29535	9	Paranoid type schizophrenia, in remission
29550	9	Latent schizophrenia, unspecified
29551	9	Latent schizophrenia, subchronic
29552	9	Latent schizophrenia, chronic
29553	9	Latent schizophrenia, subchronic with acute exacerbation
29554	9	Latent schizophrenia, chronic with acute exacerbation
29555	9	Latent schizophrenia, in remission
29580	9	Other specified types of schizophrenia, unspecified
29581	9	Other specified types of schizophrenia, subchronic
29582	9	Other specified types of schizophrenia, chronic
29583	9	Other specified types of schizophrenia, subchronic with acute exacerbation
29584	9	Other specified types of schizophrenia, chronic with acute exacerbation
29585	9	Other specified types of schizophrenia, in remission
29590	9	Unspecified schizophrenia, unspecified
29591	9	Unspecified schizophrenia, subchronic
29592	9	Unspecified schizophrenia, chronic
29593	9	Unspecified schizophrenia, subchronic with acute exacerbation
29594	9	Unspecified schizophrenia, chronic with acute exacerbation
29595	9	Unspecified schizophrenia, in remission
29600	9	Bipolar I disorder, single manic episode, unspecified
29601	9	Bipolar I disorder, single manic episode, mild
29602	9	Bipolar I disorder, single manic episode, moderate
29603	9	Bipolar I disorder, single manic episode, severe, without mention of psychotic behavior
29604	9	Bipolar I disorder, single manic episode, severe, specified as with psychotic behavior
29605	9	Bipolar I disorder, single manic episode, in partial or unspecified remission
29606	9	Bipolar I disorder, single manic episode, in full remission
29640	9	Bipolar I disorder, most recent episode (or current) manic, unspecified
29641	9	Bipolar I disorder, most recent episode (or current) manic, mild
29642	9	Bipolar I disorder, most recent episode (or current) manic, moderate
29643	9	Bipolar I disorder, most recent episode (or current) manic, severe, without mention of psychotic behavior
29644	9	Bipolar I disorder, most recent episode (or current) manic, severe, specified as with psychotic behavior
29645	9	Bipolar I disorder, most recent episode (or current) manic, in partial or unspecified remission
29646	9	Bipolar I disorder, most recent episode (or current) manic, in full remission
29660	9	Bipolar I disorder, most recent episode (or current) mixed, unspecified

29661	9	Bipolar I disorder, most recent episode (or current) mixed, mild
29662	9	Bipolar I disorder, most recent episode (or current) mixed, moderate
29663	9	Bipolar I disorder, most recent episode (or current) mixed, severe, without mention of psychotic behavior
29664	9	Bipolar I disorder, most recent episode (or current) mixed, severe, specified as with psychotic behavior
29665	9	Bipolar I disorder, most recent episode (or current) mixed, in partial or unspecified remission
29666	9	Bipolar I disorder, most recent episode (or current) mixed, in full remission
2967	9	Bipolar I disorder, most recent episode (or current) unspecified
29680	9	Bipolar disorder, unspecified
29689	9	Other bipolar disorders
30000	9	Anxiety state, unspecified
30002	9	Generalized anxiety disorder
30009	9	Other anxiety states
30921	9	Separation anxiety disorder
30924	9	Adjustment disorder with anxiety
E9287	9	Environmental and accidental causes, mechanism or component of firearm and air gun
F064	10	Anxiety disorder due to known physiological condition
F10180	10	Alcohol abuse with alcohol-induced anxiety disorder
F10280	10	Alcohol dependence with alcohol-induced anxiety disorder
F10980	10	Alcohol use, unspecified with alcohol-induced anxiety disorder
F12180	10	Cannabis abuse with cannabis-induced anxiety disorder
F12280	10	Cannabis dependence with cannabis-induced anxiety disorder
F12980	10	Cannabis use, unspecified with anxiety disorder
F13180	10	Sedative, hypnotic or anxiolytic abuse with sedative, hypnotic or anxiolytic-induced anxiety disorder
F13280	10	Sedative, hypnotic or anxiolytic dependence with sedative, hypnotic or anxiolytic-induced anxiety disorder
F13980	10	Sedative, hypnotic or anxiolytic use, unspecified with sedative, hypnotic or anxiolytic-induced anxiety disorder
F14180	10	Cocaine abuse with cocaine-induced anxiety disorder
F14280	10	Cocaine dependence with cocaine-induced anxiety disorder
F14980	10	Cocaine use, unspecified with cocaine-induced anxiety disorder
F15180	10	Other stimulant abuse with stimulant-induced anxiety disorder
F15280	10	Other stimulant dependence with stimulant-induced anxiety disorder
F15980	10	Other stimulant use, unspecified with stimulant-induced anxiety disorder
F16180	10	Hallucinogen abuse with hallucinogen-induced anxiety disorder
F16280	10	Hallucinogen dependence with hallucinogen-induced anxiety disorder
F16980	10	Hallucinogen use, unspecified with hallucinogen-induced anxiety disorder
F18180	10	Inhalant abuse with inhalant-induced anxiety disorder
F18280	10	Inhalant dependence with inhalant-induced anxiety disorder
F18980	10	Inhalant use, unspecified with inhalant-induced anxiety disorder
F19180	10	Other psychoactive substance abuse with psychoactive substance-induced anxiety disorder
F19280	10	Other psychoactive substance dependence with psychoactive substance-induced anxiety disorder
F19980	10	Other psychoactive substance use, unspecified with psychoactive substance-induced anxiety disorder
F20	10	Schizophrenia
F200	10	Paranoid schizophrenia
F201	10	Disorganized schizophrenia
F202	10	Catatonic schizophrenia
F203	10	Undifferentiated schizophrenia
F205	10	Residual schizophrenia
F208	10	Other schizophrenia
F2089	10	Other schizophrenia
F209	10	Schizophrenia, unspecified
F250	10	Schizoaffective disorder, bipolar type
F31	10	Bipolar disorder
F310	10	Bipolar disorder, current episode hypomanic
F311	10	Bipolar disorder, current episode manic without psychotic features
F3110	10	Bipolar disorder, current episode manic without psychotic features, unspecified
F3111	10	Bipolar disorder, current episode manic without psychotic features, mild
F3112	10	Bipolar disorder, current episode manic without psychotic features, moderate
F3113	10	Bipolar disorder, current episode manic without psychotic features, severe
F312	10	Bipolar disorder, current episode manic severe with psychotic features
F316	10	Bipolar disorder, current episode mixed
F3160	10	Bipolar disorder, current episode mixed, unspecified
F3161	10	Bipolar disorder, current episode mixed, mild
F3162	10	Bipolar disorder, current episode mixed, moderate
F3163	10	Bipolar disorder, current episode mixed, severe, without psychotic features
F3164	10	Bipolar disorder, current episode mixed, severe, with psychotic features

F317	10	Bipolar disorder, currently in remission
F3170	10	Bipolar disorder, currently in remission, most recent episode unspecified
F3171	10	Bipolar disorder, in partial remission, most recent episode hypomanic
F3172	10	Bipolar disorder, in full remission, most recent episode hypomanic
F3173	10	Bipolar disorder, in partial remission, most recent episode manic
F3174	10	Bipolar disorder, in full remission, most recent episode manic
F3177	10	Bipolar disorder, in partial remission, most recent episode mixed
F3178	10	Bipolar disorder, in full remission, most recent episode mixed
F318	10	Other bipolar disorders
F3181	10	Bipolar II disorder
F3189	10	Other bipolar disorder
F319	10	Bipolar disorder, unspecified
F40	10	Phobic anxiety disorders
F408	10	Other phobic anxiety disorders
F409	10	Phobic anxiety disorder, unspecified
F41	10	Other anxiety disorders
F410	10	Panic disorder (episodic paroxysmal anxiety)
F411	10	Generalized anxiety disorder
F413	10	Other mixed anxiety disorders
F418	10	Other specified anxiety disorders
F419	10	Anxiety disorder, unspecified
F4322	10	Adjustment disorder with anxiety
F53	10	Mental and behavioral disorders associated with the puerperium, not elsewhere classified
F930	10	Separation anxiety disorder of childhood
V110	9	Personal history of schizophrenia
V400	9	Mental and behavioral problems with learning
V401	9	Mental and behavioral problems with communication (including speech)
Z865	10	Personal history of mental and behavioral disorders
Z8659	10	Personal history of other mental and behavioral disorders

Supplementary Table 2 Detailed medications of antidepressants, antipsychotics, and Z drugs.

**Antidepressants**	
Selective Serotonin Reuptake Inhibitors (SSRIs)	citalopram, escitalopram, fluoxetine, sertraline, paroxetine, and fluvoxamine
Other antidepressants except SSRIs	
Monoamine oxidase inhibitors (I-MAOs)	isocarboxazid, nialamide, phenelzine, rasagiline, selegiline, and tranylcypromine
Tricyclics (TCAs) and tetracyclics (TeCAs)	amitriptyline, amoxapine, clomipramine, desipramine, doxepine, imipramine, maprotiline, nortriptyline, protriptyline, and trimipramine
Serotonin-noradrenaline reuptake inhibitors (SNRIs)	desvenlafaxine, duloxetine, levomilnacipran, milnacipran, and venlafaxine
Serotonin partial agonist and reuptake inhibitors (SPARIs)	nefazodone and trazodone
Noradrenergic and specific serotoninergic antidepressants (NASSAs)	mirtazapine
Noradrenaline and dopamine reuptake inhibitors (NDRIs)	buproprion
**Antipsychotics**	
First-generation antipsychotics (FGAs)	chlorpromazine, fluphenazine, haloperidol, loxapine, molindone, perphenazine, promazine and trifluoperazine
Second-generation antipsychotics (SGAs)	aripiprazole, asenapine, brexpiprazole, cariprazine, clozapine, iloperidone, lurasidone, olanzapine, paliperidone, quetiapine, risperidone and ziprasidone
**Z drugs**	eszopiclone, zolpidem, and zaleplon

Supplementary Table 3 The main parameters for XGBoost based IPTW model.

Method	Parameters
XGBoost-based IPTW	cumulative = FALSE,<br>priorTreatment = FALSE,<br>verbose = FALSE,<br>version = "xgboost",<br>stop.method = "es.max",<br>n.trees = 5000

Supplementary Table 4 Baseline patient characteristics in the weighted cohort.

Variables	Patients, No. (%)		P value
	SSRIs users (n = 58283.8)	Non-users (n = 70555.3)	
Age, median (IQR), y	64.0 (54.0, 74.0)	63.0 (52.0, 73.0)	<0.001
Sex			
Male	25747.2 (44.2)	33216.1 (47.1)	<0.001
Female	32536.6 (55.8)	37339.2 (52.9)	
Weight, kg	79.7 (66.4, 95.6)	79.6 (66.0, 96.7)	0.844
Charlson comorbidity index, median (IQR)	6.0 (4.0, 8.0)	5.0 (3.0, 8.0)	<0.001
SAPS II, median (IQR)	34.0 (26.0, 43.0)	35.0 (26.0, 44.0)	<0.001
SOFA, median (IQR)	2.0 (0.0, 4.0)	1.0 (0.0, 4.0)	<0.001
Admission type			
Emergency	29934.1 (51.4)	38800.5 (55.0)	<0.001
Urgent	12697.3 (21.8)	14474.8 (20.5)	
Other	15652.4 (26.9)	17280.1 (24.5)	
First care unit			
Medical/surgical ICU	39301.9 (67.4)	49285.4 (69.9)	<0.001
Other	18982.0 (32.6)	21269.9 (30.1)	
Delirium during ICU stay	22914.8 (39.3)	31817.5 (45.1)	<0.001
Coma during ICU stay	20175.0 (34.6)	28347.4 (40.2)	<0.001
Therapy at baseline			
Antibiotic	36995.5 (63.5)	45475.8 (64.5)	0.154
Vasopressors	12290.8 (21.1)	18057.8 (25.6)	<0.001
CRRT	591.8 (1.0)	1241.3 (1.8)	<0.001
Invasive mechanical ventilation	19818.0 (34.0)	25408.7 (36.0)	0.003
Coexisting conditions			
Depression	37540.2 (64.4)	43569.1 (61.8)	<0.001
Cerebrovascular disease	8173.0 (14.0)	11697.2 (16.6)	<0.001
Dementia	1478.8 (2.5)	3303.3 (4.7)	<0.001
Hypertension	23457.5 (40.2)	28378.6 (40.2)	0.971
Myocardial infarct	9116.4 (15.6)	11928.1 (16.9)	0.009
Congestive heart failure	20869.5 (35.8)	21681.3 (30.7)	<0.001
Chronic pulmonary disease	21414.4 (36.7)	23555.5 (33.4)	<0.001
Diabetes mellitus	18024.6 (30.9)	21717.7 (30.8)	0.818
Renal disease	13715.9 (23.5)	14858.3 (21.1)	<0.001
Liver disease	9702.4 (16.6)	10563.1 (15.0)	0.001
Cancer	6384.2 (11.0)	8123.4 (11.5)	0.190
Sepsis	34371.9 (59.0)	45160.0 (64.0)	<0.001
Vital signs, median (IQR)			
Heart rate (beats/min)	86.0 (76.0, 102.0)	89.0 (77.0, 105.0)	<0.001
Mean arterial pressure (mm Hg)	82.0 (71.0, 93.0)	84.0 (72.0, 96.0)	<0.001
Respiratory rate (breaths/min)	18.0 (15.0, 23.0)	19.0 (16.0, 23.0)	<0.001
Body temperature (°C)	36.8 (36.5, 37.1)	36.8 (36.5, 37.1)	0.011
SpO2 (%)	98.0 (95.0, 100.0)	98.0 (95.0, 100.0)	<0.001
Laboratory tests, median (IQR)			
White blood cell (k/uL)	10.7 (7.8, 14.9)	10.7 (7.5, 15.0)	1.000
Hemoglobin (g/dL)	10.2 (8.8, 11.8)	10.5 (8.9, 12.1)	<0.001
Platelet (k/uL)	203.0 (146.0, 274.0)	200.0 (145.0, 267.0)	0.005
Potassium (mEq/L)	4.1 (3.7, 4.5)	4.1 (3.7, 4.6)	0.022
Sodium (mEq/L)	138.0 (135.0, 141.0)	139.0 (136.0, 141.0)	<0.001
Chloride (mEq/L)	103.0 (99.0, 107.0)	104.0 (99.0, 108.0)	<0.001
Bicarbonate (mEq/L)	24.0 (21.0, 27.0)	23.0 (20.0, 26.0)	<0.001
Glucose (mg/dL)	124.0 (101.0, 159.0)	127.0 (103.0, 163.0)	<0.001
Creatinine (mg/dL)	0.9 (0.7, 1.4)	0.9 (0.7, 1.5)	0.103
Urea nitrogen (mg/dL)	18.0 (12.0, 30.0)	19.0 (12.0, 32.0)	<0.001
Medications			
Other antidepressants	7283.6 (12.5)	7529.0 (10.7)	<0.001
Antipsychotics	3481.8 (6.0)	3476.6 (4.9)	<0.001
Z drugs	924.0 (1.6)	842.8 (1.2)	<0.001
Outcomes			
In-hospital mortality	7574.1 (13.0)	8183.1 (11.6)	0.002
Length of hospital stay (days)	8.3 (5.0, 16.0)	10.0 (5.4, 19.6)	<0.001

Supplementary Table 5 Variance inflation factor to examine the presence of multicollinearity among variables.

**Variables**	**VIF**	
	**Original cohort**	**Matched cohort**
SSRI	1.062308	1.038912
Age	1.151448	1.124067
Sex	1.014164	1.027549
Admission type	1.027495	1.034581
First care unit	1.055648	1.061723
Charlson comorbidity index	1.155166	1.124724
SAPS II	1.377853	1.332499
SOFA	1.312720	1.287504
Depression	1.027837	1.035675
Sepsis	1.227416	1.206820
Delirium	1.134399	1.099080
Coma	1.239579	1.207018
Antibiotic	1.168788	1.147235
Vasopressors	1.227499	1.194588
Invasive mechanical ventilation	1.170120	1.191271
Other antidepressants	1.069373	1.033429
Antipsychotics	1.048766	1.058064
Z drugs	1.013374	1.033644

Supplementary Table 6 Association between pre-ICU use of SSRIs and in-hospital mortality mediated by delirium and coma.

Mediator		Effect (95% CI)	SE	Z	P value
Delirium	Indirect (ab)	0.002 (-0.000, 0.004)	0.001	1.426	0.154
	Direct (c′)	0.011 (0.002, 0.022)	0.005	2.011	0.044
	Total (c)	0.012 (0.004, 0.023)	0.005	2.266	0.023
Coma	Indirect (ab)	0.002 (0.000, 0.004)	0.001	1.867	0.062
	Direct (c′)	0.011(0.002, 0.021)	0.005	2.017	0.044
	Total (c)	0.013 (0.004, 0.023)	0.005	2.344	0.019

Adjusted for age, sex, admission type, first care unit, Charlson comorbidity index, depression, sepsis, delirium, coma, SAPS II, SOFA, antibiotic, vasopressors, invasive mechanical ventilation, antipsychotics, Z drugs, and other antidepressants.

Supplementary Table 7 Baseline patient characteristics in continuing and stopping SSRIs groups.

Variables	Patients, No. (%)		P value
	Continuing SSRIs (n=759)	Stopping SSRIs (n=1473)	
Age, median (IQR), y	66.0 (57.0, 76.0)	65.0 (54.0, 74.0)	0.001
Sex			
Male	326 (43.0)	646 (43.9)	0.716
Female	433 (57.0)	827 (56.1)	
Weight, kg	79.0 (66.3, 95.3)	80.0 (67.0, 95.9)	0.412
Charlson comorbidity index, median (IQR)	6.0 (4.0, 8.0)	6.0 (4.0, 8.0)	0.835
SAPS II, median (IQR)	35.0 (28.0, 44.0)	31.0 (24.0, 39.0)	<0.001
SOFA, median (IQR)	2.0 (1.0, 4.0)	1.0 (0.0, 3.0)	<0.001
Admission type			
Emergency	308 (40.6)	740 (50.2)	<0.001
Urgent	189 (24.9)	229 (15.5)	
Other	262 (34.5)	504 (34.2)	
First care unit			
Medical/surgical ICU	360 (47.4)	1000 (67.9)	<0.001
Other	399 (52.6)	473 (32.1)	
Delirium during ICU stay	248 (32.7)	281 (19.1)	<0.001
Coma during ICU stay	335 (44.1)	279 (18.9)	<0.001
Therapy at baseline			
Antibiotic	601 (79.2)	804 (54.6)	<0.001
Vasopressors	177 (23.3)	158 (10.7)	<0.001
CRRT	7 (0.9)	12 (0.8)	0.985
Invasive mechanical ventilation	332 (43.7)	255 (17.3)	<.0001
Coexisting conditions			
Depression	603 (79.4)	1163 (79.0)	0.829
Cerebrovascular disease	87 (11.5)	204 (13.8)	0.128
Dementia	21 (2.8)	57 (3.9)	0.222
Hypertension	346 (45.6)	603 (40.9)	0.039
Myocardial infarct	150 (19.8)	233 (15.8)	0.022
Congestive heart failure	289 (38.1)	478 (32.5)	0.009
Chronic pulmonary disease	288 (37.9)	543 (36.9)	0.650
Diabetes mellitus	249 (32.8)	465 (31.6)	0.585
Renal disease	158 (20.8)	371 (25.2)	0.025
Liver disease	116 (15.3)	183 (12.4)	0.070
Cancer	70 (9.2)	194 (13.2)	0.008
Sepsis	407 (53.6)	515 (35.0)	<0.001
Vital signs, median (IQR)			
Heart rate (beats/min)	82.0 (76.0, 96.0)	84.0 (73.0, 99.0)	0.823
Mean arterial pressure (mm Hg)	80.0 (70.0, 91.0)	82.0 (71.0, 93.0)	0.013
Respiratory rate (breaths/min)	17.0 (15.0, 22.0)	18.0 (15.0, 22.0)	0.006
Body temperature (°C)	36.7 (36.4, 37.0)	36.8 (36.4, 37.1)	<0.001
SpO2 (%)	99.0 (96.0, 100.0)	97.0 (95.0, 100.0)	<0.001
Laboratory tests, median (IQR)			
White blood cell (k/uL)	11.2 (8.0, 15.3)	10.0 (7.2, 13.7)	<0.001
Hemoglobin (g/dL)	9.5 (8.3, 10.9)	10.3 (8.9, 11.8)	<0.001
Platelet (k/uL)	181.0 (132.0, 242.5)	205.0 (150.0, 277.0)	<0.001
Potassium (mEq/L)	4.2 (3.8, 4.6)	4.1 (3.7, 4.5)	0.094
Sodium (mEq/L)	138.0 (136.0, 140.0)	138.0 (136.0, 141.0)	0.855
Chloride (mEq/L)	105.0 (100.0, 109.0)	103.0 (99.0, 107.0)	<0.001
Bicarbonate (mEq/L)	23.0 (21.0, 26.0)	24.0 (22.0, 27.0)	<0.001
Glucose (mg/dL)	123.0 (103.0, 150.0)	122.0 (101.0, 153.0)	0.771
Creatinine (mg/dL)	0.9 (0.7, 1.2)	0.9 (0.7, 1.4)	0.611
Urea nitrogen (mg/dL)	17.0 (13.0, 28.0)	18.0 (12.0, 30.0)	0.847
Medications			
Other antidepressants	239 (31.5)	357 (24.2)	<0.001
Antipsychotics	103 (13.6)	174 (11.8)	0.260
Z drugs	36 (4.7)	49 (3.3)	0.124
Outcomes			
In-hospital mortality	108 (14.2)	81 (5.5)	<0.001
Length of hospital stay (days)	6.7 (4.4, 11.0)	5.8 (3.9, 9.1)	<.0001

Supplementary Figure 1 The proportion of missing data for variables in the original cohort.

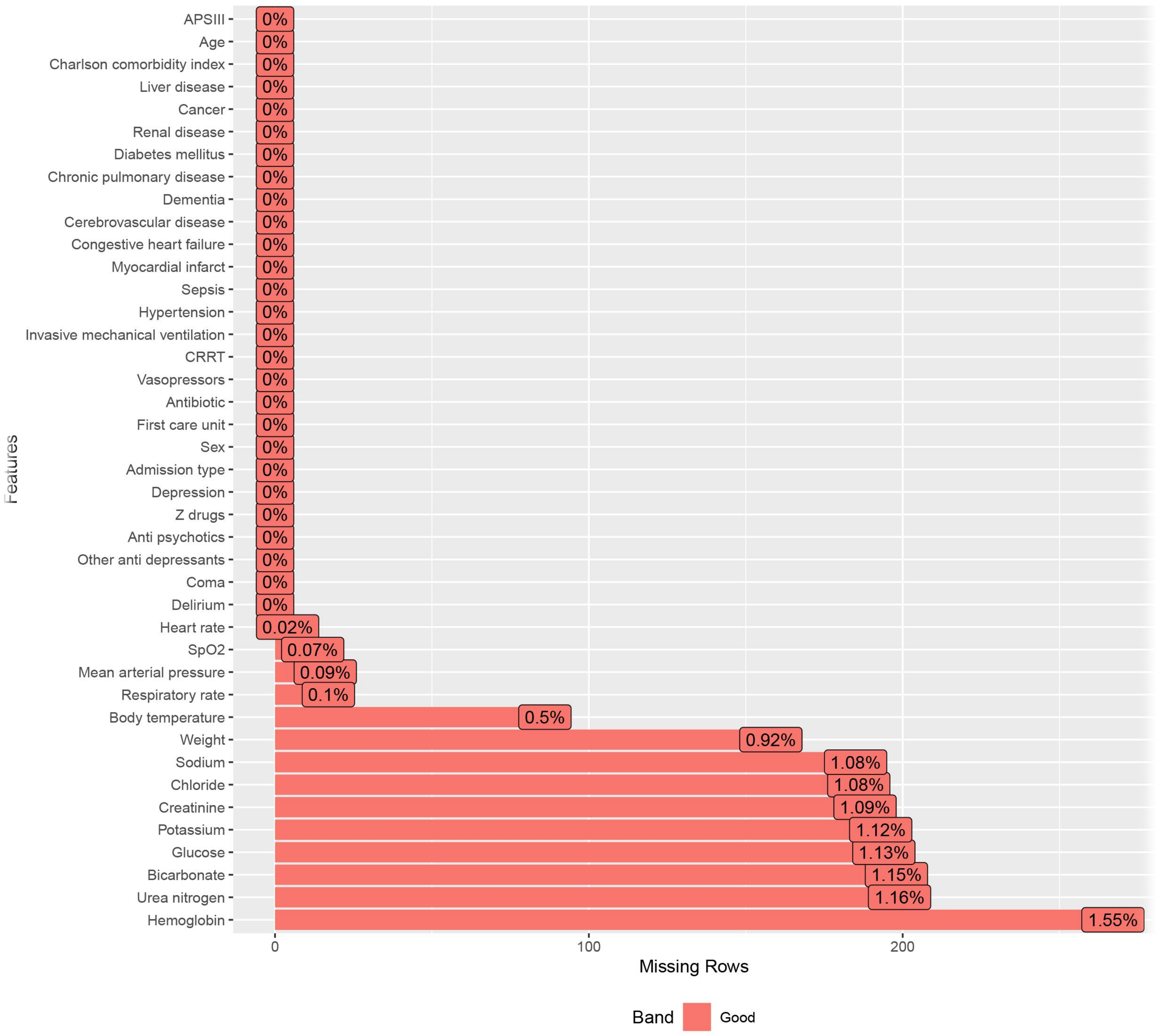

Supplementary Figure 2 Standardized mean differences in variables for the original cohort (red squares), the matched cohort (green circles), and the weighted cohort (blue triangles).

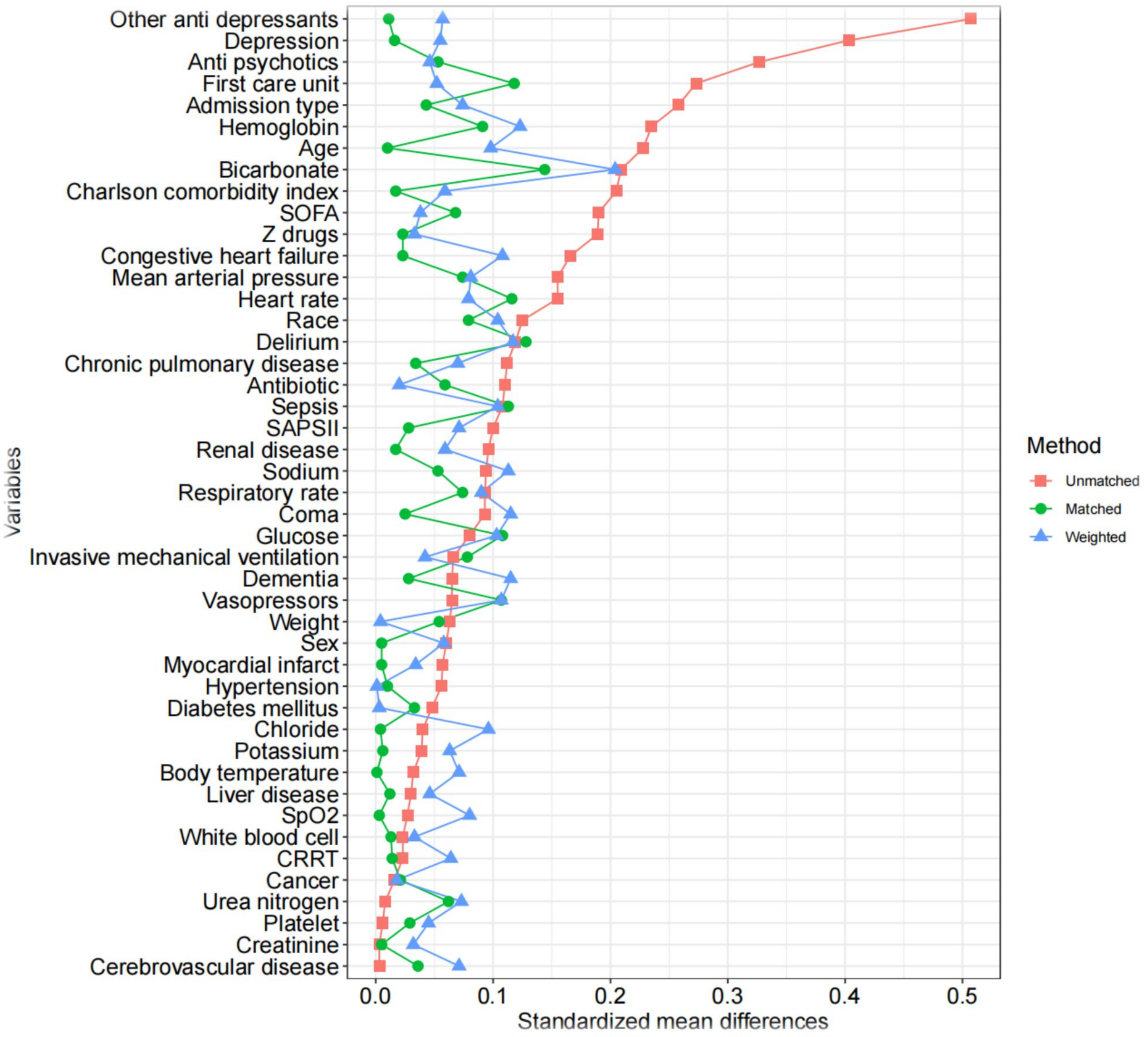

Supplementary Figure 3 Schoenfeld residual test to check the assumption of proportional hazard in the original cohort.

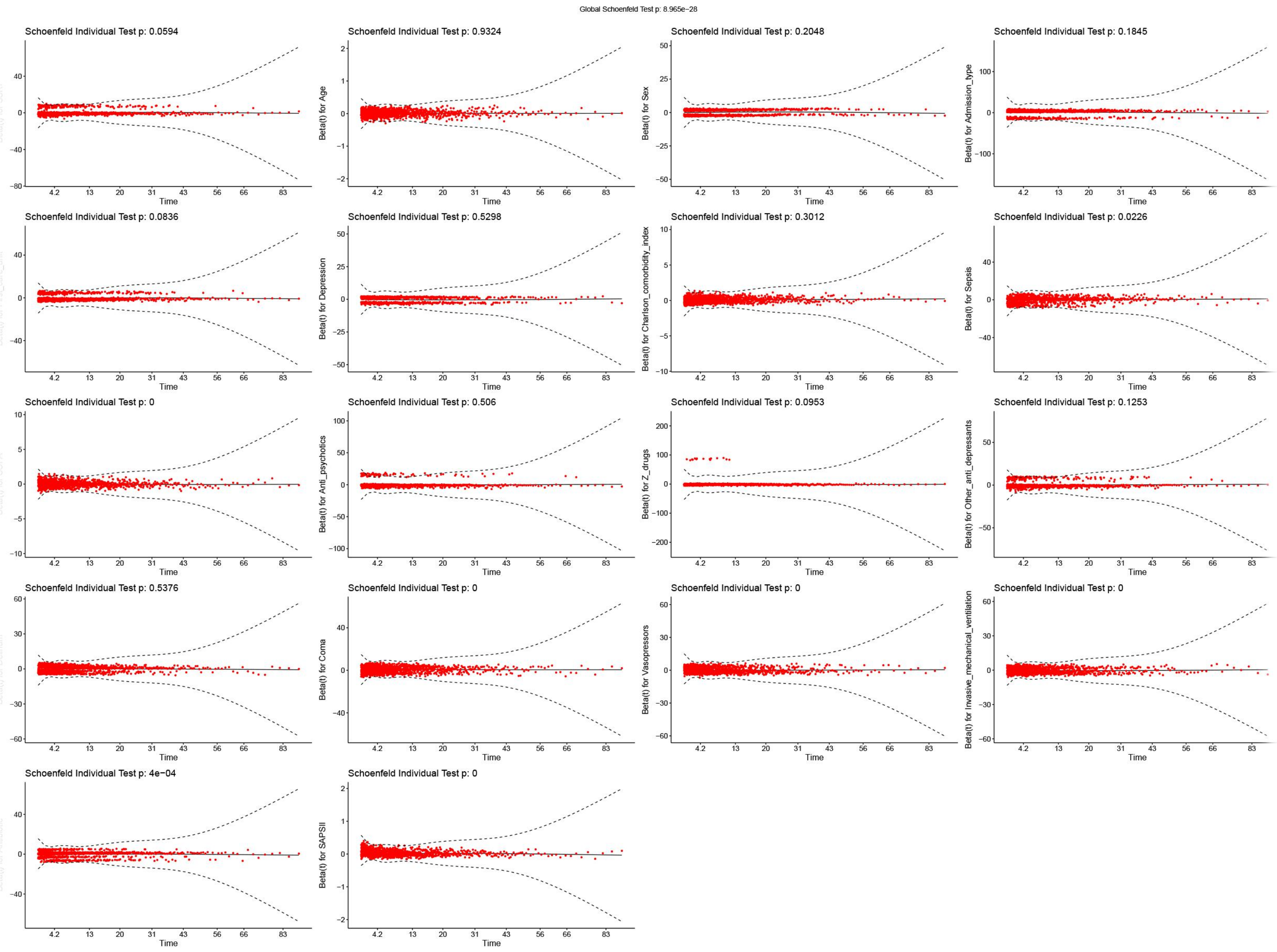

Supplementary Figure 4 Schoenfeld residual test to check the assumption of proportional hazard in the matched cohort.

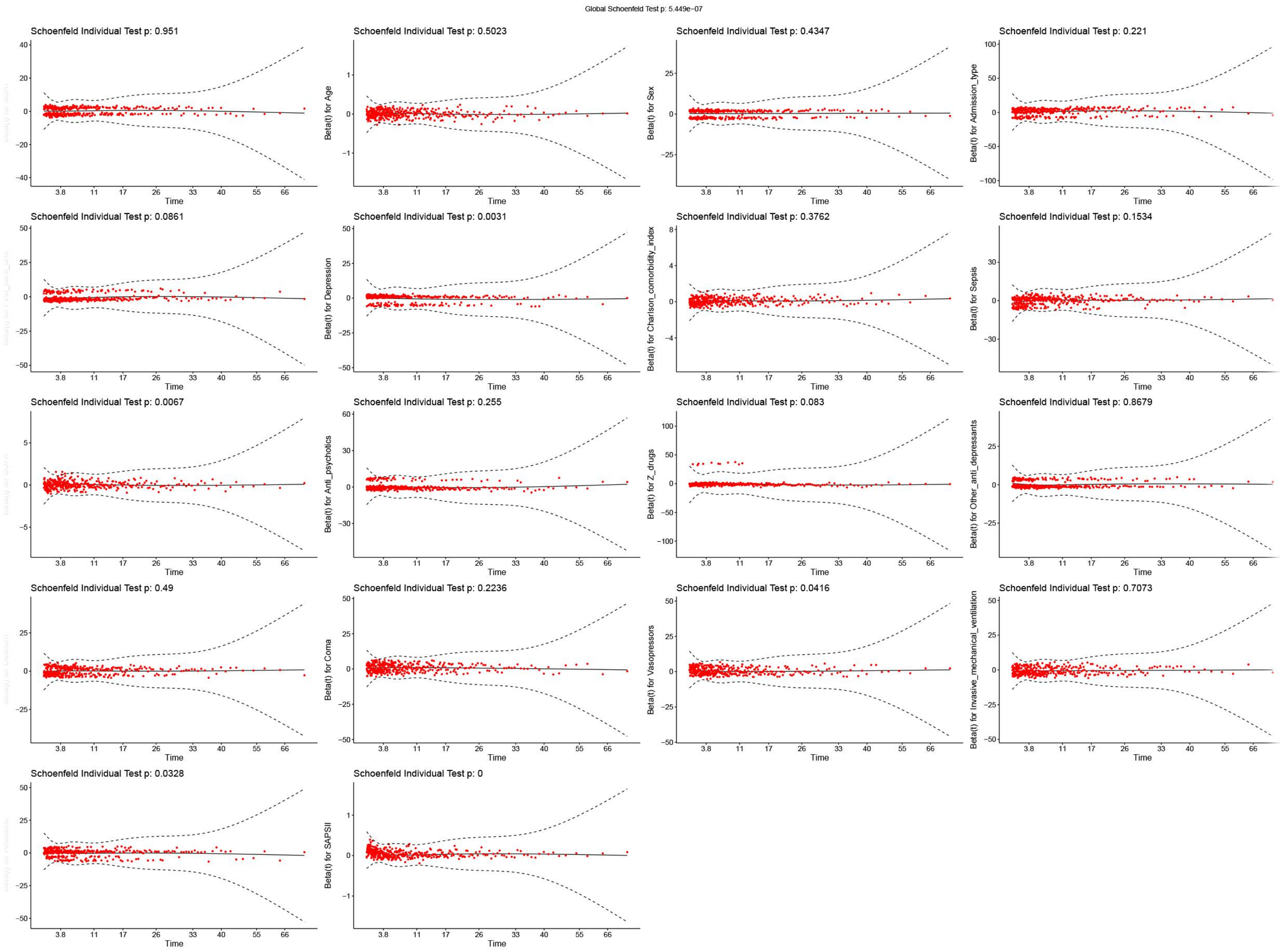

Supplementary Figure 5 Density function showing the distribution balance for the variables before and after matching.

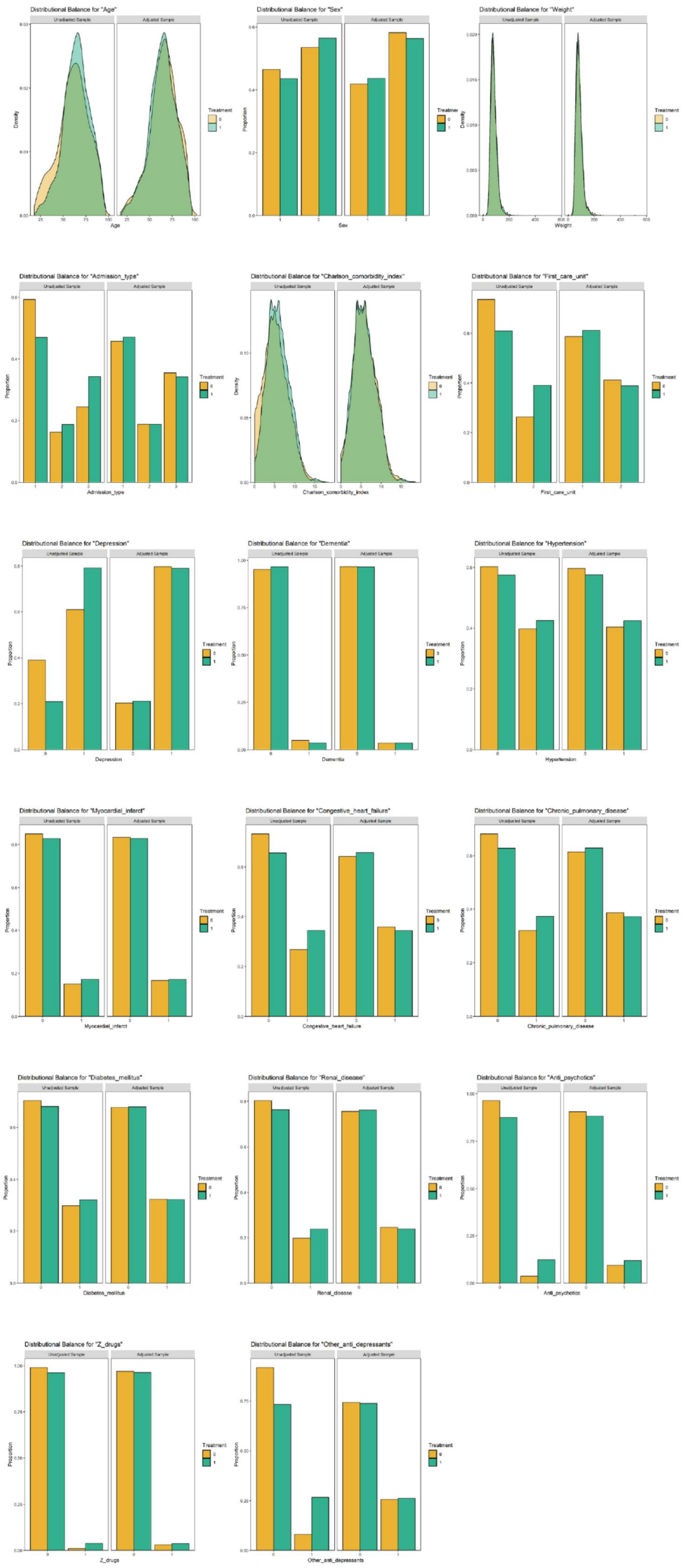

Supplementary Figure 6 Density function showing the distribution balance for the variables before and after weighting.

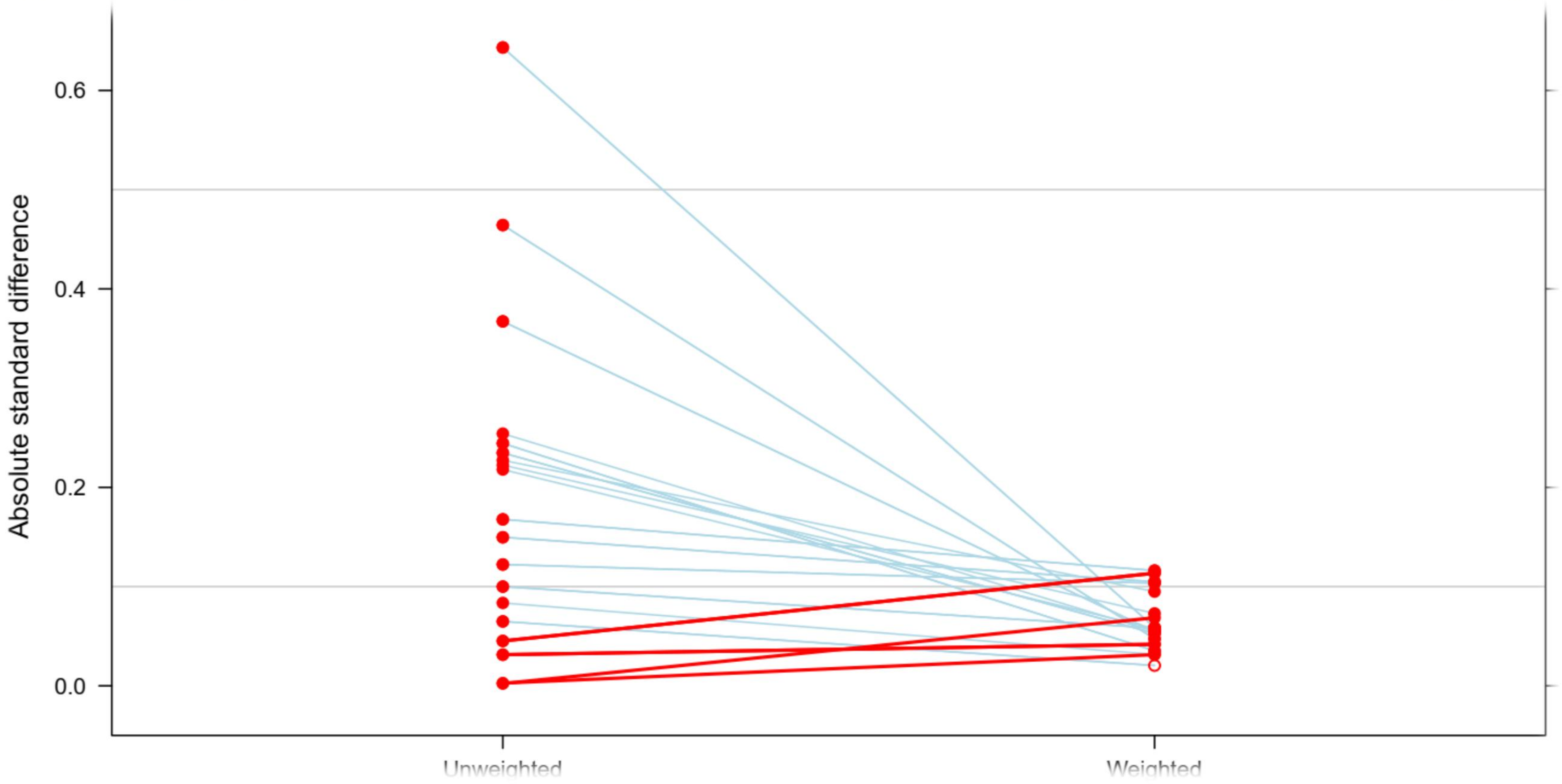

Supplementary Figure 7 Plot of the standardized effect size of the variables before and after weighting.

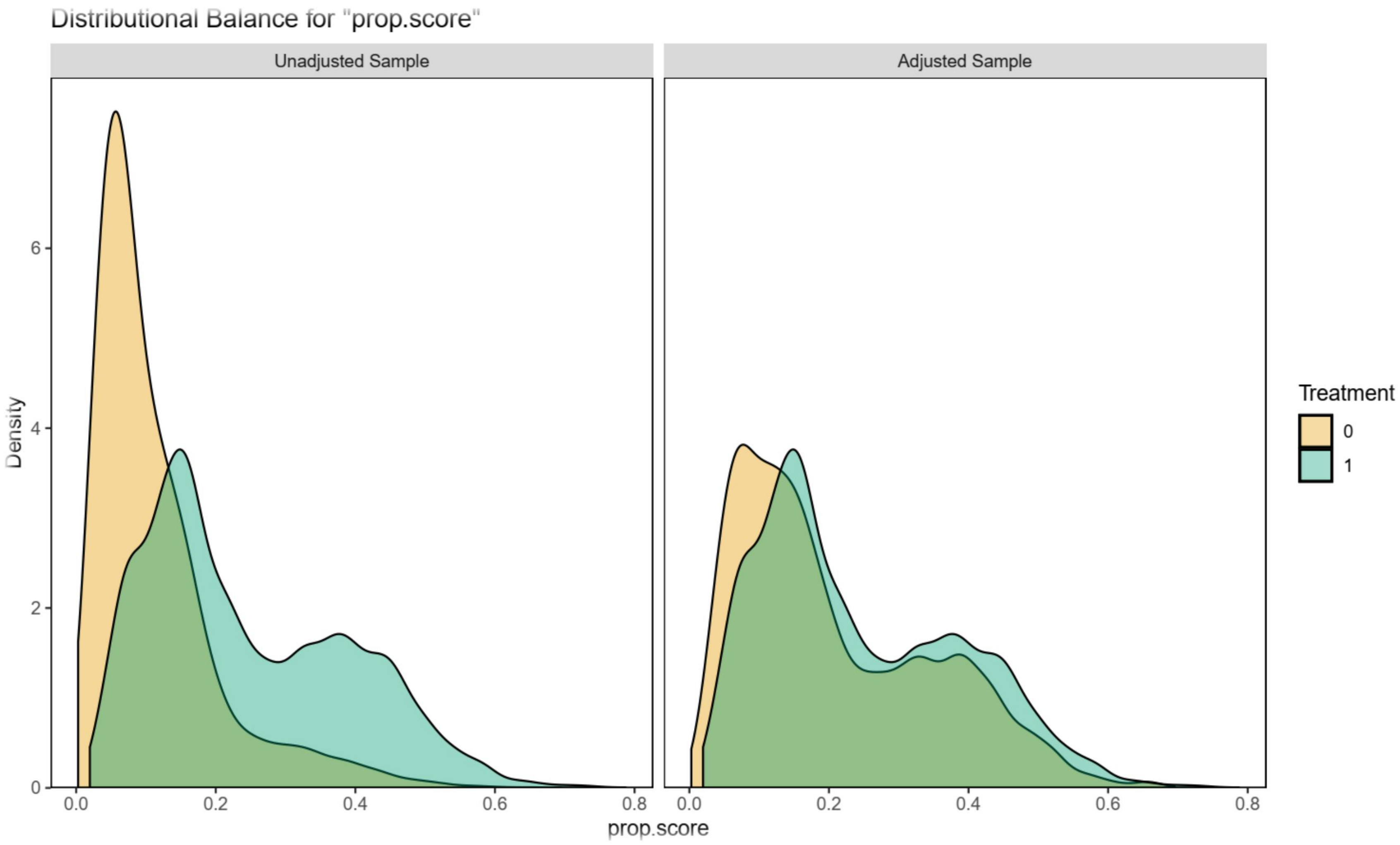

Supplementary Figure 8 Forest plot for the class effect analysis.

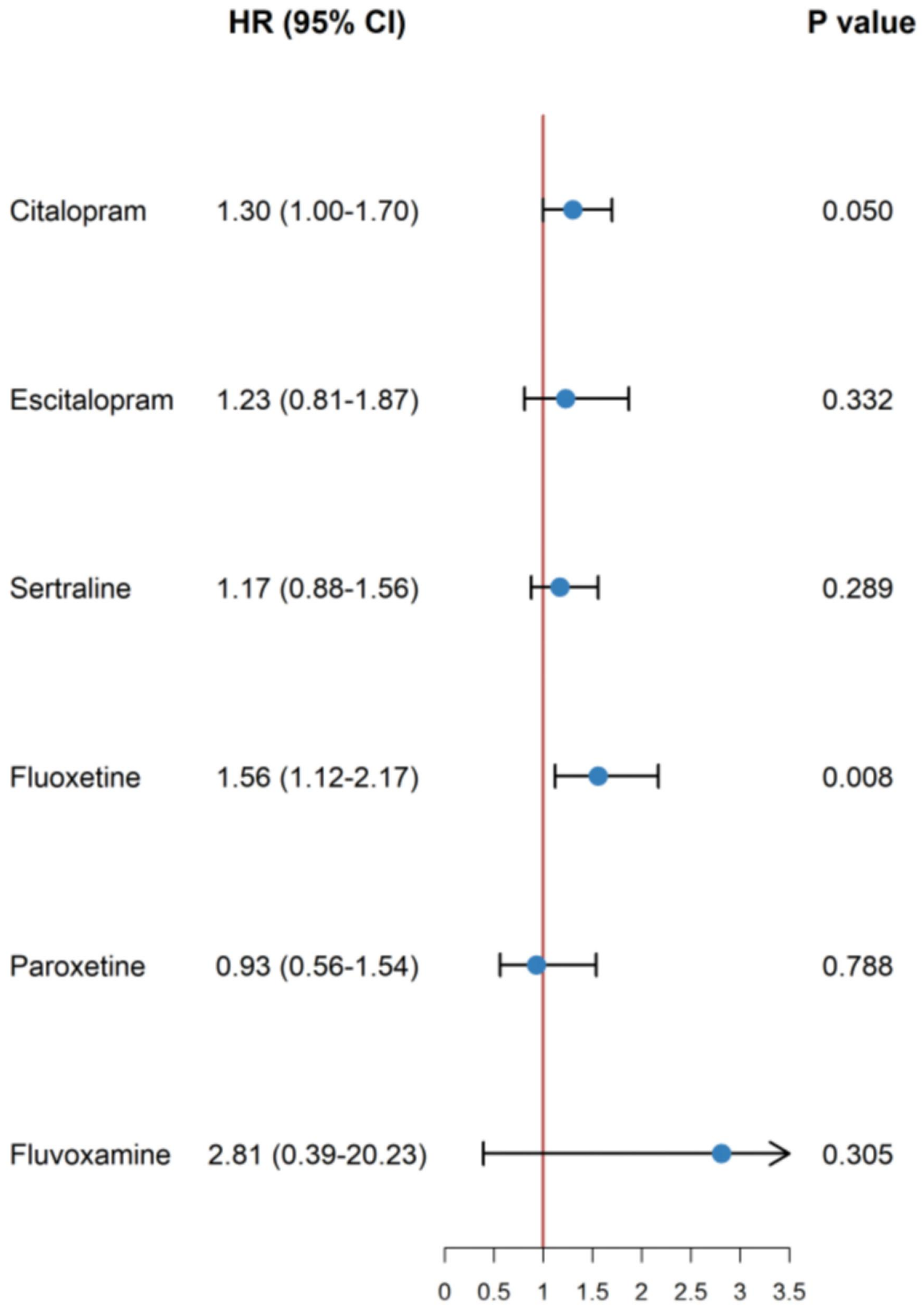

Supplement: Supplementary file 1 — Supplemental Material [file 41398_2023_2487_MOESM1_ESM.pdf]
